# Single-Center Outcomes of Epstein–Barr Virus DNAemia in Adult Solid Organ Transplant Recipients

**DOI:** 10.1155/2024/5598324

**Published:** 2024-01-18

**Authors:** Sara W. Dong, Barbra M. Blair, Carolyn D. Alonso

**Affiliations:** ^1^Division of Infectious Disease, Department of Medicine, Beth Israel Deaconess Medical Center, Harvard Medical School, Boston, MA, USA; ^2^Division of Infectious Disease, Department of Pediatrics, Boston Children's Hospital, Harvard Medical School, Boston, MA, USA

## Abstract

**Background:**

Immunosuppression in solid organ transplantation (SOT) increases the risk of Epstein–Barr virus (EBV) DNAemia, which may herald development of posttransplant lymphoproliferative disease (PTLD). Few studies have characterized the incidence, risk factors, and clinical impact of EBV DNAemia in adult SOT recipients (SOTR).

**Methods:**

A single-center, retrospective review of adult (≥18 years) SOTR between 01 January 2015 and 31 December 2019 was conducted. Patients were stratified by the primary study endpoint of development of EBV DNAemia (whole blood EBV DNA PCR > 200 copies/mL). Secondary endpoints included development of PTLD, reduction in immunosuppression (RIS), use of pre-emptive therapy, and all-cause mortality.

**Results:**

Among 442 adult SOTR, the predominant transplant organs were the kidney (258, 58%) and liver (141, 31.9%). EBV serostatus in most subjects (430, 97%) was classified as intermediate risk (R+). Eight subjects (2%) were high risk (donor (D+/R−), and 4 (1%) were low risk (D−/R−). The overall incidence of EBV DNAemia was 4.1% (18/442) with a median time to detection of 14 months (range 3–60). The highest proportion of DNAemia was observed in D+/R− subjects (37.5%; *p* < 0.001). Development of PTLD was significantly associated with EBV DNAemia and occurred in 3/18 patients with DNAemia (16.7%) vs. 3/424 (0.7%) without DNAemia (*p* < 0.001). All patients with PTLD were managed with RIS and rituximab.

**Conclusion:**

We observed that EBV D+/R− serostatus and development of sustained EBV DNAemia were high risk features associated with subsequent development of PTLD in our cohort of adult SOTR.

## 1. Introduction

Advances in immunosuppressive agents have reduced the incidence of transplant organ rejection and prolonged allograft and patient survival, but patients' susceptibility to opportunistic infection has increased. The risk of infection in a transplant recipient is impacted by epidemiologic exposures and the net state of immunosuppression and can involve viruses (such as cytomegalovirus (CMV), Epstein–Barr virus (EBV)), bacteria (Gram-positive, Gram-negative, and mycobacteria), fungi/molds (such as *Candida* and *Aspergillus* spp.), and parasites (such as *Toxoplasma*). Immunosuppression in solid organ transplantation (SOT) increases the risk of Epstein–Barr virus (EBV) DNAemia, which may herald development of posttransplant lymphoproliferative disease (PTLD). PTLD remains one of the most significant complications of SOT [1]. EBV plays a predominant role in early PTLD within the first year of transplant and is found in the majority of early diagnoses [1–5]. Previously noted risk factors for both EBV DNAemia and PTLD have relied on single-center studies as well as larger registry datasets [2–4, 6, 7]. The most notable risk factor for both EBV DNAemia and PTLD has been donor/recipient mismatch of EBV serostatus (donor seropositive, recipient seronegative; D+/R−) [1, 6, 7]. Other risk factors for EBV DNAemia posttransplant include antithymocyte globulin (ATG) induction treatment as well as CMV mismatch [6, 8–10]. The incidence of PTLD varies by the organ transplanted with small intestinal transplant recipients at highest risk with rates up to 32%. Pancreas, heart, lung, and liver transplants are moderate risk groups (rates between 3 and 12%), while renal transplant confers a lower risk (1-2%) [1–3, 7].

Given the high proportion of EBV-positive PTLD in the first year after transplant, quantitative serum EBV PCR testing has become a routine test for monitoring transplant recipients who are considered high risk for developing PTLD. Guidelines currently recommend screening for EBV DNA in high-risk recipients for one year after transplantation, but the optimal strategy for this surveillance is unclear [1]. There are no clear viral load thresholds that are known to correlate with the development of PTLD [11–14]. It is also difficult to translate values across laboratories due to lack of consensus in calibration, specimen type, and unit of reporting [15]. Pre-emptive prevention strategies typically combine serial quantitative EBV DNA monitoring with interventions before the onset of clinical diseases in high-risk patients [1, 2, 4, 16]. Few studies have described the epidemiology of EBV DNAemia in adult recipients. Therefore, we examined a cohort of adult SOT recipients to characterize the incidence and clinical impact of EBV DNAemia. We hypothesized that elevated and sustained EBV DNAemia would be associated with PTLD.

## 2. Materials and Methods

### 2.1. Study Design and Data Collection

We conducted a single-center retrospective review of adult (≥18 years of age) SOT recipients at a single academic center between 01 January 2015 and 31 December 2019. For patients who received multiple transplants within the study period, the most recent transplant was used for analysis. All patients had a minimum of 15 months of follow-up. Patients were censored by the date of death or the last clinic follow-up. Donor demographic and transplant information was available as part of the Ottr® Organ transplant management software system (CareDx, Omaha, NE). Recipient demographic information, transplant care, outcomes, immunosuppression, allograft rejection, viral data, physical exam, and pathology and radiology reports were extracted from the medical record. Only biopsy-proven acute rejection episodes were included, and rejection was defined according to the Banff classification [17, 18].

Study data were collected and managed using REDCap electronic data capture tools [19, 20]. The primary study endpoint was the development of EBV DNAemia. Secondary endpoints included the development of PTLD as defined by WHO criteria, reduction in immunosuppression (RIS), use of pre-emptive therapy (e.g., anti-CD20 agent rituximab), and all-cause mortality [21].

At this center, the standard immunosuppression protocol for kidney and pancreas transplantations includes 3-4 doses of rabbit antithymocyte globulin, 5-day corticosteroid taper, and initiation of calcineurin inhibitors and mycophenolate. Basiliximab is utilized if the recipient's age ≥70 years or two haplotype match live donor kidney. The standard protocol for liver transplantations includes a corticosteroid taper (85 days) with calcineurin inhibitors and mycophenolate. Basiliximab is considered for patients with kidney failure immediately prior to transplant.

### 2.2. Viral Serology and Quantification of EBV DNA

The presence of anti-EBV viral capsid antigen IgG was determined using a commercially available enzyme immunoassay (EIA) on recipients' sera as pretransplantation work-up and on donor sera available at transplantation. Whole blood quantitative EBV DNA PCRs were assayed (Quest Diagnostics Nichols Institute, Chantilly, VA). The lower limit of detection of the assay is <200 copies/mL, <2.30 Log copies/mL. Based on PCR results, patients were classified as having no DNAemia or EBV DNAemia (EBV DNA >200 copies/mL). Sustained EBV DNAemia was then defined as the presence of EBV DNA detection (>200 copies/mL) on at least 3 consecutive samples. The duration of EBV DNAemia was based on time from the first positive EBV PCR result to the last positive result with no intervening negative results.

At our center, current institutional recommendations for monitoring and treatment of EBV DNAemia after transplant are based on pre-emptive or symptom-driven screening (such as unexplained fevers, drenching sweats, weight loss, adenopathy, sore throat or oral ulcers, unexplained cytopenia, or elevated transaminases/creatinine). Routine EBV PCR from SOT recipients is not recommended unless the patient is high-risk serostatus (D+R−). EBV naive recipients are recommended to have EBV PCR at least monthly for 6 months and every 3 months through year 1. These local recommendations were revised in 2019 towards the end of the study period though, so EBV results in this study were obtained as part of clinical care.

### 2.3. Statistical Analysis

Clinical characteristics were summarized with descriptive statistics, and univariate analysis of the associations between donor, recipient, and graft variables and the development of EBV DNAemia was performed. The EBV DNAemia and no EBV DNAemia groups were compared using the Mann–Whitney test for continuous variables and the chi-square test or Fishers exact test for categorical variables. *p* values <0.05 were considered statistically significant. Kaplan–Meier curves were plotted describing the time to first development of EBV DNAemia and subdivided by baseline EBV serostatus.

Statistical analyses were performed using Stata/IC 16.1 (StataCorp LLC, College Station, TX)

### 2.4. Ethics Statement

The study was approved by the Institutional Review Board of our center (IRB No. 2020P000744). Informed consent requirement was waived by the Board.

## 3. Results

### 3.1. Study Population

The study included 442 adult SOT recipients. Patient demographics and transplant characteristics are shown in Table 1. The median age at transplant was 55 years (range 23–74 years) and 314 (71%) were men. The predominant transplant organ was the kidney (*n* = 258; 58.4%), followed by the liver (*n* = 141; 31.9%), kidney-pancreas (*n* = 22; 5.0%), kidney-liver (*n* = 15; 3.4%), and pancreas (*n* = 6; 1.4%). Recipient EBV serostatus was known for all included patients; 97% of the entire cohort was classified as EBV intermediate risk (R+). High-risk (D+/R−) status accounted for 8 patients (1.8%), and the remaining were low risk (D−R−) (*n* = 4; 1.0%).

### 3.2. EBV DNAemia Characteristics

**Figure 1:**
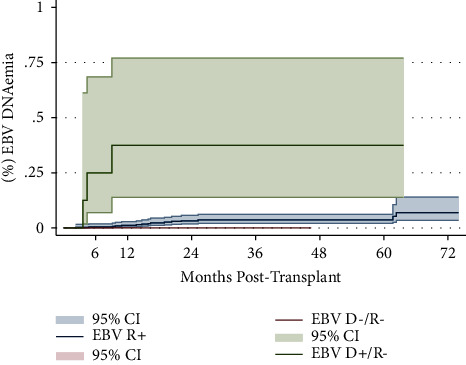
Incidence of EBV DNAemia in SOTR by EBV serostatus. Proportions of SOTR with EBV DNAemia by first time to detection posttransplant, compared to EBV serostatuses of EBV intermediate risk R+, EBV low risk D−/R−, and EBV high risk D+/R− (*p* value <0.001; log-rank test). EBV, Epstein–Barr virus; SOTR, solid organ transplant recipient; R, recipient; D, donor; CI, confidence interval.

Within the entire cohort, 265 patients did not have EBV DNA PCR collected (60.0%). Of the 177 patients (40%) who had EBV DNA PCR testing performed, 18 SOT recipients (4.1% of all included patients) had detectable EBV PCR results on at least one occasion. The median number of available EBV PCR results in these 18 patients was 5 (range: 2–38). Among those who developed DNAemia, the median viral load was 1956 copies/mL (range 214−1.9 million copies/mL), and the median time to first EBV detection was 14 months (range 3–60 months). Eight (1.8%) subjects had sustained DNAemia. The median viral load in the sustained DNAemia group was 2573 copies/mL, and the median time to DNAemia was shorter at 10 months. Time to development of first detectable DNAemia is illustrated in Figure 1, compared to EBV serostatus. EBV DNAemia occurred earlier posttransplant in the D+/R− group (median 9 months) compared to those in the R+ group (median 16 months), *p* value <0.001.

### 3.3. Factors and Outcomes Associated with EBV DNAemia

Univariate analysis of factors associated with the development of EBV DNAemia is shown in Table 1. Patients with and without DNAemia did not differ based on age, sex, transplant organ, previous transplant, CMV serostatus, or presence of rejection. EBV DNAemia was more likely to be observed in the D+/R− group (3/8, 37.5%) compared to subjects without DNAemia (15/430, 3.5%; *p* < 0.001). There was no difference demonstrated in induction or maintenance immunosuppression regimens.

Eighty-two (18.6%) of all 442 patients had an episode of rejection during the follow-up period. Five patients had evidence of both EBV DNAemia and rejection: 2 patients with rejection preceding EBV DNAemia >1 year, 1 patient with concurrent rejection and DNAemia, and 2 patients with rejection after episode of EBV DNAemia (5 and 10 months).

There was no significant difference in mortality between the EBV DNAemia and no EBV DNAemia groups, and 96.4% of the entire cohort was alive at 1-year posttransplant. PTLD was identified in 6 subjects (1.4%). Of patients with PTLD, 50% had evidence of DNAemia preceding the diagnosis and all were sustained DNAemia. All patients with PTLD were managed with RIS and receipt of rituximab. Detailed clinical characteristics of the patients who developed PTLD are noted in Table 2.

## 4. Discussion

Clinical guidelines recommend EBV surveillance in all seronegative mismatch recipients on a weekly to biweekly basis, while surveillance is not routinely recommended for R+ SOT recipients with the exception of intestinal transplant [1]. Studies on the use of quantitative EBV surveillance in SOT are overall limited with data primarily from pediatric transplant recipients due to higher rates of EBV R− status in that population [1, 14, 15, 22, 23]. The incidence of EBV DNAemia in the first year posttransplant has previously been reported within a wide range of 13–67% in adult SOT recipients. Additionally, sustained EBV DNAemia has been observed at rates up to 72% in adult liver and 30% in adult kidney recipients [6, 8–10, 24–26]. The large degree of variation is likely related to variability in immunosuppression regimens and nonstandardized tests. Optimal management of EBV DNAemia posttransplant is also not clear. Reduction in immunosuppression (RIS) remains the mainstay, but there is no clear consensus on the use of anti-CD20 agents such as rituximab.

In this study, the primary endpoint of interest was the development of EBV DNAemia. The overall incidence of EBV DNAemia in this adult transplant cohort was 4.1%. We found the highest proportion of EBV DNAemia in subjects who were D+/R− at time of transplant (37.5%) compared with rates of 3.5% and 0% in the R+ and D−/R− groups, respectively. The overall rate of EBV DNAemia in this study is lower than in previous reports in adult SOT recipients (13–67%) [6, 8–10, 24–26]. Most recently, Blazquez-Navarro et al. observed 18.4% of patients in a multicenter cohort experienced EBV reactivation in the first posttransplant year [6]. The infrequent screening in our cohort may have led to underestimation of rates of EBV DNAemia. Only 40% of subjects had EBV monitoring during the study. A substantial proportion of D+/R− subjects (3/8, 37.5%) did not have EBV screening performed. No differences were identified in rates of EBV DNAemia according to immunosuppression, including ATG induction [6, 8].

Secondary endpoints focused on presence and management of PTLD and all-cause mortality. While uncommon, the development of PTLD in our cohort was significantly associated with EBV DNAemia and occurred in 3/18 patients with DNAemia (16.7%) compared to 3/424 (0.7%) in those without DNAemia (*p* value <0.001). All patients with PTLD were managed with RIS and rituximab. Within the patients with EBV DNAemia, rituximab was used in 4/18 total patients (22%). Mortality was low in the study with 96% of patients alive at 1 year with no difference based on presence of EBV DNAemia.

There are limitations to this study. First, this was a retrospective, single-center study. The incidence of EBV DNAemia was too low to perform multivariate analysis for further identification of possible risk factors of EBV DNAemia. Second, the pattern of EBV sampling was not standardized within the cohort, but this does demonstrate the overall low rate of EBV surveillance, which is not surprising, given the predominantly intermediate to low-risk population typically seen in adult transplant recipients. Third, we followed patients for a minimum of 15 months; therefore, our study is unable to address the incidence of late onset EBV DNAemia.

Despite these limitations, we provide an analysis of a large adult cohort with details on the real-world management of patients with EBV DNAemia and PTLD at our center. Quantitative EBV PCR monitoring in SOT recipients remains a potentially important intervention to research further. Implementation at our center of standardized processes for monitoring EBV DNAemia in the highest-risk patients would provide more insights into the incidence of DNAemia, which we found was significantly associated with the risk of PTLD in this cohort. Additional research is needed to examine the use of EBV DNAemia for PTLD risk stratification and whether any adjunctive laboratory tests may improve specificity of high viral load as a predictor. Viral load dynamics/kinetics [14] might be a practical way to predict the development of PTLD in clinical settings. Last, future research directions include the evaluation of patients with persistent sustained viral load as the clinical significance of the chronic viral load phenotype is unclear.

## Figures and Tables

**Table 1:** Characteristics of solid organ transplant recipients according to development of EBV DNAemia.

Characteristics	Total (*n* = 442)	EBV DNAemia (*n* = 18)	No EBV DNAemia (*n* = 424)	*p* value
Median age at transplant, years (IQR)	55 (23–74)	54.5 (50–68)	55 (24–74)	0.47
Male, sex, *n* (%)	314 (71.0)	10 (55.6)	304 (71.2)	0.14
Transplant organ, *n* (%)				0.59
Kidney	258 (58.4)	10 (55.6)	248 (58.5)	
Liver	141 (31.9)	5 (27.8)	136 (32.1)	
Pancreas	6 (1.4)	1 (5.6)	5 (1.2)	
Kidney-liver	15 (3.4)	1 (5.6)	14 (3.3)	
Kidney-pancreas	22 (5.0)	1 (5.6)	21 (5.0)	
Previous transplant, *n* (%)	37 (8.4)	3 (16.7)	34 (8.0)	0.19
EBV serostatus at transplant, *n* (%)				<0.001
EBV low risk (D−/R−)	4 (1.0)	0	4 (1.0)	
EBV intermediate risk (R+)	430 (97.3)	15 (83.3)	415 (97.9)	
EBV high risk (D+/R−)	8 (1.8)	3 (16.7)	5 (1.2)	
CMV serostatus at transplant, *n* (%)				0.74
CMV low risk (D−/R−)	107 (24.2)	3 (16.7)	104 (24.5)	
CMV intermediate risk (R+)	239 (54.1)	11 (61.1)	228 (53.8)	
CMV high risk (D+/R−)	96 (21.7)	4 (22.2)	92 (21.7)	
Induction immunosuppression, *n* (%)				
Basiliximab	37 (8.4)	2 (11.1)	35 (8.4)	0.67
Polyclonal antilymphocyte antibodies	279 (63.1)	11 (61.1)	268 (62.3)	0.86
Steroids^†^	127 (28.7)	5 (27.8)	122 (28.8)	0.93
Maintenance immunosuppression, *n* (%)				
Cyclosporine	7 (1.6)	1 (5.6)	6 (1.4)	0.17
Tacrolimus	435 (98.4)	17 (94.4)	418 (98.6)	0.17
Azathioprine	1 (0.2)	0	1 (0.2)	0.84
Mycophenolate	436 (98.6)	18 (100)	418 (98.6)	0.61
Sirolimus	4 (0.9)	0	4 (0.94)	0.68
Belatacept	1 (0.2)	0	1 (0.2)	0.84
Prednisone <20 mg/day	30 (6.8)	2 (11.1)	28 (6.6)	0.46
Prednisone (≥20 mg/day)	6 (1.4)	0	6 (1.4)	0.61
Episode of rejection, *n* (%)	82 (18.6)	5 (27.8)	77 (18.2)	0.30
Clinical outcomes, *n* (%)				
Development of PTLD	6 (1.4)	3 (16.7)	3 (0.7)	<0.001
Reduction in immunosuppression	6 (1.4)	3 (16.7)	3 (0.7)	<0.001
Receipt of anti-CD20 agent	6 (1.4)	3 (16.7)	3 (0.7)	<0.001
Receipt of chemotherapy^‡^	3 (0.1)	1 (5.6)	2 (0.1)	0.01
No development of PTLD	436 (98.6)	15 (83.3)	421 (99.3)	
Reduction in immunosuppression	2 (0.4)	2 (11.1)	0	<0.001
Receipt of anti-CD20 agent	1 (0.2)	1 (5.6)	0	<0.001
Alive at 1 year	426 (96.4)	17 (94.4)	409 (96.4)	0.65

EBV, Epstein–Barr virus; CMV, cytomegalovirus; IQR, interquartile range. ^†^≥1 g methylprednisolone or equivalent. ^‡^Chemotherapy regimens for these three patients included (1) etoposide, prednisone, vincristine, cyclophosphamide, doxorubicin, and rituximab (DA-EPOCH); (2) rituximab, cyclophosphamide, doxorubicin, vincristine, and prednisone (R-CHOP); and (3) R-CHOP and later EPOCH with bortezomib.

**Table 2:** Clinical characteristics of adult solid organ transplant recipients who developed PTLD.

Case no.	Tx year	Sex/age	Organ	Underlying disease	EBV serostatus	Time from transplant to		Peak titer of EBV load (copies/mL)	Median titer of EBV load (copies/mL)	Involved PTLD sites/primary site	Histologic diagnosis (pathologic findings)	Treatment (rituximab^†^, RIS, chemotherapy^‡^)	Clinical outcome (PTLD, graft loss/function)	Alive at 1 year
						EBV DNAemia	PTLD diagnosis							
1	2019	21/M	Kidney	Nephronophthisis	D+/R−	4 months	Early 4 months	796	229	Bilateral palatine and adenoid tonsils; cervical lymph nodes; and spleen	Early lesions (infectious mononucleosis-like) +EBER	RIS	Complete response	Yes

2	2017	64/F	Liver	Primary biliary cirrhosis	D+/R+	N/A	Early 3 months	N/A	N/A	Liver allograft; porta hepatis, portocaval, retrocaval, and mesenteric lymph nodes	Polymorphic or monomorphic, unclear which subtype −EBER	RIS Rituximab	Complete response	Yes

3	2016	63/M	Liver	Alcoholic/HCV cirrhosis	D+/R+	N/A	Late 23 months	N/A	N/A	Liver allograft; posterolateral spleen; and retrocrural lymph nodes	Monomorphic/Burkitt's lymphoma EBER	RIS Rituximab chemo	Complete response	Yes

4	2016	58/M	Liver	Alcoholic cirrhosis	D+/R−	3 months	Early 7 months	1,964,383	16,112	Liver allograft; adrenal gland; axillary and mediastinal LAD	Polymorphic +EBER	RIS Rituximab	Complete response	Yes

5	2015	53/M	Liver	NASH cirrhosis; HCC	D+/R+	N/A	Late 32 months	N/A	N/A	Cervical, axillary, mediastinal, R hilar lymph nodes; multiple subpleural pulm nodules; and liver allograft	Monomorphic/diffuse large B-cell lymphoma −EBER	RIS Rituximab chemo	Complete response	Yes

6	2015	68/M	Kidney	Diabetes mellitus type 2	D+/R−	8 months	Early 9 months	124,693	4,597	Submandibular mass; lung; and upper abdominal/pancreatic mass	Monomorphic/diffuse large B-cell lymphoma +EBER	RIS Rituximab chemo	Partial response	No, death at 11 months

Tx, transplant; EBV, Epstein–Barr virus; PTLD, posttransplant lymphoproliferative disease; D, donor; R, recipient; N/A, not applicable; EBER, Epstein–Barr virus-encoded small RNA; RIS, reduction in immunosuppression; chemo, chemotherapy; pulm, pulmonary. ^†^Rituximab regimens included induction therapy (4 doses administered weekly) followed by consolidative therapy (3-4 doses administered every 3 weeks). ^‡^Chemotherapy regimens for these three patients included (case no. 3) etoposide, prednisone, vincristine, cyclophosphamide, doxorubicin, and rituximab (DA-EPOCH); (case no. 5) rituximab, cyclophosphamide, doxorubicin, vincristine, and prednisone (R-CHOP); (case no. 6) R-CHOP and later EPOCH with bortezomib.

## Data Availability

The data used to support the findings of this study are available from the corresponding author upon request.

## Abbreviations

EBV: Epstein–Barr virus
SOT: Solid organ transplantation
PTLD: Posttransplant lymphoproliferative disease
D/R: Donor/recipient
ATG: Antithymocyte globulin
CMV: Cytomegalovirus.
